# Recurrent Pericarditis and Paradigm Shift in Cardiovascular Imaging and Targeted Therapeutics

**DOI:** 10.1016/j.jacadv.2024.101194

**Published:** 2024-09-18

**Authors:** Rehan Karmali, Tahir S. Kafil, Aqieda Bayat, Bianca Honnekeri, Osamah Badwan, Felix Berglund, Paul Cremer, Allan L. Klein

**Affiliations:** a Center for Diagnosis and Treatment of Pericardial Diseases, Section of Cardiovascular Imaging, Robert and Suzanne Tomsich Department of Cardiovascular Medicine, Sydell and Arnold Miller Family Heart, Vascular and Thoracic Institute, Cleveland Clinic, Cleveland, Ohio, USA; b Bluhm Cardiovascular Institute, Northwestern University Feinberg School of Medicine, Chicago, Illinois, USA

**Keywords:** autoinflammatory phenotype, interleukin-1, cardiac magnetic resonance, multimodality imaging, recurrent pericarditis

## Abstract

Recurrent pericarditis poses a significant challenge to patients and clinicians given its high morbidity and health care burden. Since the last iteration of European Society of Cardiology Guidelines in 2015, further insights have been gained into the pathophysiology, multimodality imaging assessment, and treatment of this condition. The purpose of this review is to discuss each of these aspects and highlight the role of imaging-guided therapy and interleukin-1 inhibitors in autoinflammatory phenotypes that together have transformed the care of these patients. Although future investigations are needed to optimize diagnostic surveillance and timing of therapy, recent evidence points at an encouraging paradigm shift in the treatment of recurrent pericarditis.

## Highlights

• Imaging-guided therapy with cardiac magnetic resonance imaging allows for assessment of pericardial edema and inflammation, constrictive physiology, prognostication, and can guide therapy.
• Recurrent pericarditis often manifests as an autoinflammatory phenotype characterized by pericardial late gadolinium enhancement or elevated serum inflammatory markers.
• Multiple randomized trials provide evidence of safety and efficacy of IL-1 blockers in recurrent pericarditis.
• There is a new paradigm shift in the diagnosis and therapeutics in patients with recurrent pericarditis.

## Case

A 35-year-old male with Cushingoid appearance presents with a second recurrence of pleuritic chest pain that radiates to the trapezius muscles and is relieved by leaning forward. His first bout of acute pericarditis was 8 months earlier for which he was started on high-dose nonsteroidal anti-inflammatory drugs (NSAIDs) and colchicine. His symptoms initially resolved, but he returned 4 months later due to an episode of 9/10 pleuritic chest pain with elevated ultrasensitive C-reactive protein (CRP) of 18.9 mg/L and electrocardiogram showing widespread ST-segment elevations and PR depressions. He was diagnosed with idiopathic recurrent pericarditis and prednisone 50 mg daily was added. He was instructed to taper his prednisone by 5 mg every 2 weeks while continuing NSAIDs and colchicine, but presents now 15 weeks after his first recurrence with symptoms as noted. A repeat echocardiogram showed a moderate circumferential pericardial effusion and cardiac magnetic resonance (CMR) imaging showed moderate pericardial late gadolinium enhancement (LGE) and pericardial edema. He was diagnosed with a second recurrence of pericarditis and considered to be steroid-dependent and colchicine-resistant. What diagnostic work-up should be pursued? How should his treatment regimen be escalated? What is the modern approach to recurrent pericarditis?

Patients with recurrent pericarditis represent a unique population that has significant morbidity as a result of intractable symptoms and medication side effects. Up to one-third of patients with acute pericarditis experience multiple recurrences,^1^ and pericarditis recurrence is associated with a near doubling of all-cause health care costs along and significant quality of life impairments.^2^ Recent clinical trials have enhanced our understanding of idiopathic recurrent pericarditis as an autoinflammatory disease. Multimodality imaging including CMR has been fundamental to characterize disease severity and facilitate imaging-guided therapy. Clinicians have targeted tools at their disposal now when treating a patient with recurrent pericarditis in 2024 compared to 2015 when the last comprehensive European Society of Cardiology guidelines were formulated. As such, the purpose of this review is to propose a paradigm shift in recurrent pericarditis and outline new therapies that can help reduce the overall morbidity and economic burden of this disease.

## Pathophysiology

**Figure 1:**
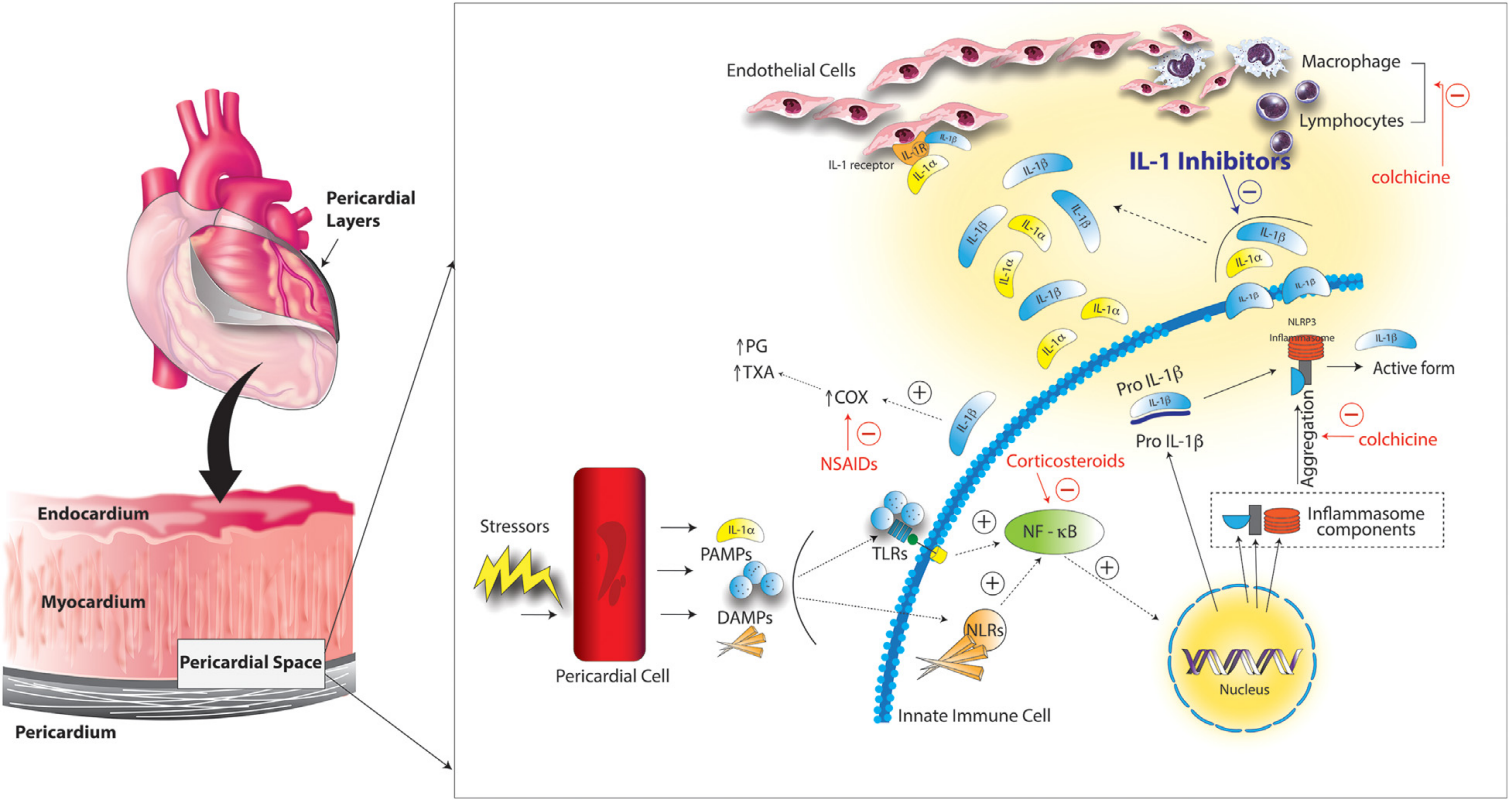
**Pathophysiology of the Autoinflammatory Phenotype of Recurrent Pericarditis** An acute injury to mesothelial cells of the pericardium can activate the innate immune system via interleukin-1 (IL-1α), pathogen-associated molecular patterns (PAMPs) and damage-associated molecular patterns (DAMPs). These activate toll-like receptors (TLRs) and nuclear factor light chain enhancer (NF-B) and enhance activity of the nucleotide-binding oligomerization domain-like receptor pyrin domain-containing (NLRP3) inflammasome. Subsequently, the effector caspase cleaves the pro-inflammatory cytokine pro- IL-1β into its active form. Both IL-1α and IL-1β bind to IL-1 receptors in capillary and endothelial cells leading to an influx of neutrophils, macrophages, and monocytes and amplification of pericardial inflammation. Adapted with permission from Kumar et al.^16^ COX = cyclooxygenase; NSAID = nonsteroidal anti-inflammatory drug; PG = prostaglandin; TXA = thromboxane.

The typical etiology for pericarditis in developing countries is tuberculosis and in developed countries is postviral or idiopathic.^3^^,^^4^ Postcardiac injury syndromes after myocardial infarction, coronary interventions, electrophysiology procedures, or cardiac surgery are other offending triggers along with autoimmune diseases and malignancy.^5^ Seventy to 90% of patients with recurrences have idiopathic/viral pericarditis making treatment challenging.^6^ Furthermore, those who have experienced >2 recurrences have an approximately 20% to 40% risk for having subsequent episodes.^7^ Our understanding of this disease’s pathophysiology is framed by similarities noted between recurrent pericarditis and the Periodic Fever Syndromes including genetically determined autoinflammatory diseases such as Familial Mediterranean Fever (FMF). These syndromes are characterized by unprovoked attacks of multisystem inflammation due to multiple associated mutations including a missense mutation in a pyrin coding gene. This leads to constitutive activation of nucleotide-binding oligomerization domain-like receptor pyrin domain-containing (NLRP3) inflammasome and interleukin (IL)-1, which are key effectors in the innate immune system.^8^^,^^9^ Furthermore, it is hypothesized that NLRP3 activation rather than viral infection may underlie first episodes of acute idiopathic pericarditis.^10^ This is in contrast to autoimmune diseases such as systemic lupus erythematosus where the adaptive immune system drives the disease pathology via action of antigen-specific T cells and autoantibodies.^7^^,^^11^ Activation of the innate immune system in diseases like FMF results in patients suffering from episodic bouts of fever, serositis, and arthritis lasting 1 to 3 days.^12^ Patients with recurrent pericarditis also share genetic features with IL-1-mediated autoinflammatory conditions as seen with *MEFV1* variants, a gene that plays a key role in IL-1 processing.^13^ The similarities in pathophysiology between FMF and recurrent pericarditis also relate to the effectiveness of colchicine in both diseases. Colchicine inhibits NLRP3 activation via the P2X2 and P2X7 pores and caspase 1, thereby decreasing downstream pro-inflammatory cytokine release.^14^ Recent investigations underscore the innate immune system and its effector mechanisms in the pathogenesis of recurrence in pericarditis.^15^ Figure 1 highlights the proposed pathophysiology. An acute injury to mesothelial cells of the pericardium can activate the innate immune system.^17^ The innate immune system contains inflammasomes which are activated by exogenous or endogenous signals.^18^ Human pericardial samples of patients with chronic pericarditis have shown an intensification of NLRP3 inflammasome expression.^19^ The NLRP3 inflammasome consists of a sensor, a scaffold protein, and an effector caspase.^20^^,^^21^ Exposure to a pericardial irritant leads to the formation of this inflammasome and subsequent cleavage of pro-IL-1β into its active form by caspase. IL-1β is the predominant circulating isoform of IL-1, an apical pro-inflammatory cytokine, that stimulates the synthesis of inflammatory mediators including cyclo-oxygenase-2 and prostaglandins.^11^^,^^18^ IL-1α is initially produced as a precursor and stored in the cytoplasm of cells of mesenchymal origin such as pericardial cells. The cascade of autoinflammation leads to release of IL-1α which also stimulates the transcription of IL-1β from monocytes. Both IL-1α and IL-1β bind to IL-1 receptors in capillary and endothelial cells leading to an influx of neutrophils, macrophages, and monocytes and amplification of pericardial inflammation.^15^^,^^22^

## Clinical diagnosis and multimodality imaging

The diagnostic criteria for recurrent pericarditis are similar those of acute pericarditis.^23^ It requires the initial pericardial attack and 2 out of the following 4 features: pleuritic chest pain worse with inspiration and when supine, electrocardiogram changes including ST-segment elevation or PR segment depression, a pericardial friction rub on auscultation or a new or worsening pericardial effusion.^24^ Recurrent pericarditis is further defined as having a relapse of symptoms following a symptom-free period of 4 to 6 weeks or longer after the initial episode of pericarditis.^3^ Patients with a known history of recurrent pericarditis on multiple anti-inflammatories may have some of these features masked. Hence, supportive findings such as elevated inflammatory markers (eg, high-sensitivity CRP) or imaging evidence of pericardial inflammation on CMR aid in the diagnosis.^25^ Incessant pericarditis is defined as persistent symptoms without a symptom-free period and is considered more aggressive than recurrent pericarditis.^26^

**Figure 2:**
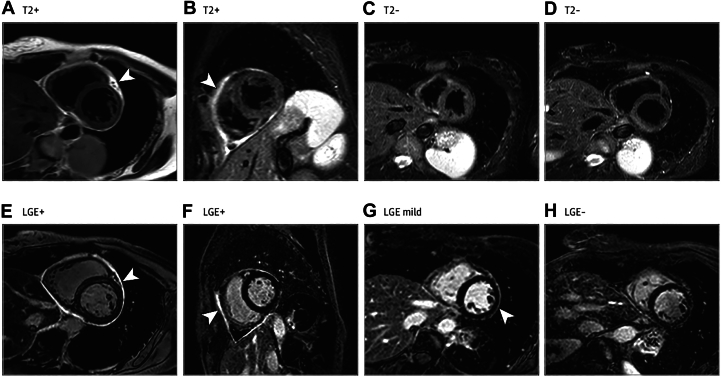
**Cardiac Magnetic Resonance Findings in Recurrent Pericarditis** Cardiac magnetic resonance showing an increased signal intensity on T2-weighted sequences representing pericardial edema (A and B) and pericardial late gadolinium enhancement (LGE) and inflammation (E and F) with interval improvement on anti-inflammatory therapy (C and G) and resolution (D and H). Adapted with permission from Kumar et al.^16^

An echocardiogram helps establish baseline cardiac function, assess for pericardial effusion, tamponade physiology, wall motion abnormalities, or features of constrictive physiology such as interventricular dependence and increased respiratory variation of Doppler flows.^27^^,^^28^ In cases of diagnostic uncertainty, CMR is a valuable modality as an imaging biomarker to ascertain morphologic and hemodynamic information, assess for myocardial involvement and provide pericardial tissue characterization (Figure 2). Black blood spin-echo sequences can highlight pericardial thickness, while bright blood cine imaging such as steady-state free precession can showcase effusions and signs of constriction.^29^^,^^30^ T2-weighted imaging is used for assessing pericardial edema, which is usually present in acute inflammation and may resolve in the subacute and chronic stage.^31^ In addition, LGE is a highly sensitive CMR finding of pericardial inflammation and represents underlying neutrophil infiltration, fibrin deposition, and neovascularization.^32^ It is challenging to interpret continued yet reduced LGE in patients on follow-up CMR imaging who are on anti-inflammatory therapy. This often implies some residual neovascularization or inflammation that improves with time with anti-inflammatories. Serial CMR studies show resolution of edema and decreased LGE in patients who have improved clinically with anti-inflammatory treatment suggesting decreased pericardial inflammation and neovascularization rather than fibrosis. Overall, the combination of pericardial LGE and pericardial edema on T2-short tau inversion recovery imaging in CMR has a high specificity for diagnosing a recurrence of pericarditis compared to the clinical criteria alone.^33^^,^^34^ The presence of a high burden of pericardial LGE can predict a shorter time to and a greater likelihood of recurrence and a longer time to achieve clinical remission.^33^^,^^34^ These findings, in combination with assessment of inflammatory markers, can help identify patients at risk for recurrence and inform treatment duration and tapering strategies.^35^^,^^36^

An imaging-guided therapy approach incorporating CMR has been shown to decrease pericarditis recurrence as well as cumulative steroid exposure.^37^ CMR also has utility in guiding therapy of patients with transient (subacute) constrictive pericarditis. Repetitive insults to the pericardium from ongoing inflammation can lead to loss of pericardial compliance. This can lead to constrictive pathophysiology seen on echocardiography with features including diastolic septal bounce and respirophasic septal shift suggestive of interventricular dependence. In addition, the mitral valve and tricuspid valve inflow can show a respiratory variation with peak E velocity >25% and 40%, respectively, increased annular medial early velocity compared to lateral velocity (annulus reversus) and increased diastolic expiratory hepatic vein flow reversal compared to forward diastolic flow.^38^ CMR can also highlight several features of constriction including pericardial thickening, abrupt cessation of diastolic filling, abnormal septal motion including respirophasic shift on free breathing sequence, inspiratory septal flattening suggestive of ventricular interdependence and pericardial tethering.^26^ Significantly increased LGE of pericardium indicates ongoing active inflammation which may be contributing to transient constriction.^39^ Treating this with anti-inflammatory agents may improve constriction.^40^ Conversely, the absence of pericardial LGE in a patient with imaging evidence of constriction and clinical features of heart failure suggests underlying fibrosis that is unlikely to respond to anti-inflammatory therapy. Cardiac computed tomography (CT) informs burden and pattern of pericardial calcification. Cardiac CT helps in planning pericardiectomy in patients with pericardial constriction unresponsive to medical therapy.^41^

## Established treatment options

Treatments for recurrent pericarditis aim to reduce inflammation and provide sustained symptom relief (Table 1). With a potentially wide array of triggers, a common cause of recurrent pericarditis is inadequate treatment of the first episode of pericarditis or inadequate tapering of medication.^42^ Hence, understanding first-line therapies is essential. NSAIDs are long considered first-line therapy for patients with pericarditis and key clinical trials in patients with post-pericardiotomy syndrome showed a high efficacy and highlighted their role.^43^ Typical initial dose of ibuprofen is 600 to 800 mg every 8 hours for a duration of weeks to months and in contemporary practice, high-dose NSAIDS such as ibuprofen combined with colchicine are highly effective in alleviating symptoms. NSAIDs are tapered following symptomatic improvement and resolution of inflammatory markers (CRP) ^23^^,^^26^ by decreasing doses by 200 to 400 mg every 2 to 4 weeks. Aspirin is an alternative to NSAIDs in patients with coexisting coronary or peripheral artery disease.^44^ However, there is no evidence to suggest that aspirin is inferior to other NSAIDs and is often preferred in some parts of the world regardless of the presence or absence of coexistent vascular disease. Lastly, gastroprotection with a proton-pump inhibitor should be emphasized. Colchicine is recommended as first-line therapy for acute and recurrent pericarditis in conjunction with aspirin/NSAIDs.^23^ Usual initial dose includes 0.6 mg (0.5 mg in Europe) 1 to 2 times daily (for patients <70 kg or intolerant to higher doses) with a treatment duration of at least 6 months. If symptoms and inflammatory markers improve, then patients may taper to 0.6 mg daily to then 0.3 mg daily and eventually discontinue the medication. Multicenter trials have demonstrated a decreased rate of pericarditis recurrence and quick and sustained remission of symptoms with colchicine use.^45^, ^46^, ^47^ Two subsequent meta-analysis reaffirmed a robust risk reduction of recurrent pericarditis with colchicine.^48^^,^^49^ Duration of therapy is initially 3 months with extension to 6 months if a recurrence occurs.^23^^,^^26^ Patients may develop intolerance due to gastrointestinal symptoms like nausea or diarrhea, in which case a dose reduction is reasonable. Low-dose corticosteroids (ie prednisone 0.2-0.5 mg/kg/d) may be added for treatment of pericarditis refractory to NSAIDs and colchicine or in situations where these medications are contraindicated or not tolerated.^23^ While steroids may provide relief of symptoms transiently, their unopposed use particularly at high doses can lead to dependence medication side effects and an increased risk of recurrence and prolonged disease course.^50^, ^51^, ^52^, ^53^ Tapering quickly is associated with risk for relapse.^53^ Hence, when clinical improvement is noted as evidenced by resolution of chest pain or CRP, steroid dose may be decreased by 5 to 10 mg every 1 to 2 weeks until a 20 mg daily dose, after which it is decreased by 2.5 mg every 1 to 2 weeks to 10 mg, then by 1 mg every 1 to 2 weeks until weaning off.^23^^,^^54^ If pericarditis recurs while tapering the steroid, then increasing background therapy with NSAIDs and colchicine first is favored as opposed to increasing steroid dose.^55^ Unfortunately, a subset of patients will have breakthrough symptoms of pericarditis despite optimal medical therapy. While azathioprine and intravenous immunoglobulin carry a class IIB recommendation as third-line agents per the 2015 European Society of Cardiology guidelines, evidence is limited to a single-center retrospective study and case series, respectively.^56^^,^^57^ Their utility may be greater in patients with concomitant autoimmune disease and steroid-related adverse effects who need to be weaned off steroids. Furthermore, exercise is not recommended in the active inflammatory stages of acute and recurrent pericarditis.^58^

**Table 1:** Medical Management for Pericarditis

Medication	Dosing and Duration	Tapering Strategy	Common Side Effects	Special Considerations
NSAIDs (ibuprofen)	Initial: 600 mg every 8 h (1-2 wk) Recurrence: 600 mg every 8 h (wk to mo)	Reduce dose by 200-400 mg every 2-4 wk following improvement of symptoms and inflammatory markers	Gastrointestinal inflammation Prolonged bleeding time Kidney injury	Use as first-line treatment for acute pericarditis and for first recurrence Use with proton pump inhibitor (PPi)
Aspirin	Initial: 750–1,000 mg every 8 h (1-2 wk) Recurrence: 500-1,000 mg every 6-8 h (wk to mo)	Reduce by 250-500 mg every 1-2 wk following improvement of symptoms and inflammatory markers	Gastrointestinal inflammation Prolonged bleeding time Kidney injury	Use as first-line treatment for acute pericarditis and first recurrence Use with proton pump inhibitor (PPi)
Colchicine	Initial: 0.6 mg (<70 kg) or 0.6 mg twice daily (>70 kg) (3 mo) Recurrence: 0.6 mg daily or twice daily for at least 6 mo	Reduce to 0.6 mg daily, then to 0.3 mg daily every 2 wk, then off following improvement of symptoms and inflammatory markers	Gastrointestinal upset—nausea, vomiting, or diarrhea	Use as first-line treatment for acute pericarditis and first recurrence Use with proton pump inhibitor (PPi)
Corticosteroids	Prednisone 0.25-0.5 mg/kg/d	Reduce by 5-10 mg every 1-2 wk following improvement of symptoms and inflammatory markers. At a dose of 20 mg daily, decrease by 2.5 mg every 1-2 wk, then by 1 mg every 1-2 wk	Hyperglycemia Weight gain Adrenal suppression Hypertension Fluid retention Psychiatric disturbances Peptic ulcer disease Infection Myopathy	Continue background therapy with NSAIDs and/or colchicine while on steroids. During a flare, increase NSAIDs/colchicine first instead of increasing steroids.
Rilonacept	Loading dose - 320 mg Maintenance: 160 mg/wk for 2 y	Limited data: individualize stopping or tapering strategy based on inflammatory markers and CMR findings	Injection site reaction Upper respiratory tract infection	Introduce background therapy with colchicine when stopping or tapering
Anakinra	1-2 mg/kg daily injection (maximum 100 mg/d) for 6-12 mo	After 6-12 mo if symptoms, LGE and inflammatory markers improved, then taper by 100 mg/wk every month till a 300 mg/cumulative weekly dose is achieved and then further reduction by 100 mg/wk every 2-3 mo	Injection site reaction Transaminase elevation	May continue background therapy with colchicine Note not FDA approved
Goflikicept	Loading dose:160 mg 1 wk later: 70 mg 1 wk later: 80 mg Then, continue 80 mg every 2 wk Total duration ∼ 10 mo	Limited data: Individualize tapering strategy based on inflammatory markers and CMR findings	Injection site reaction Upper respiratory infection Neutropenia	May continue background therapy with colchicine if still experiencing symptoms Note not FDA approved

CMR = cardiac magnetic resonance; LGE = late gadolinium enhancement; NSAID = nonsteroidal anti-inflammatory drug.

## Novel targeted therapeutics

**Figure 3:**
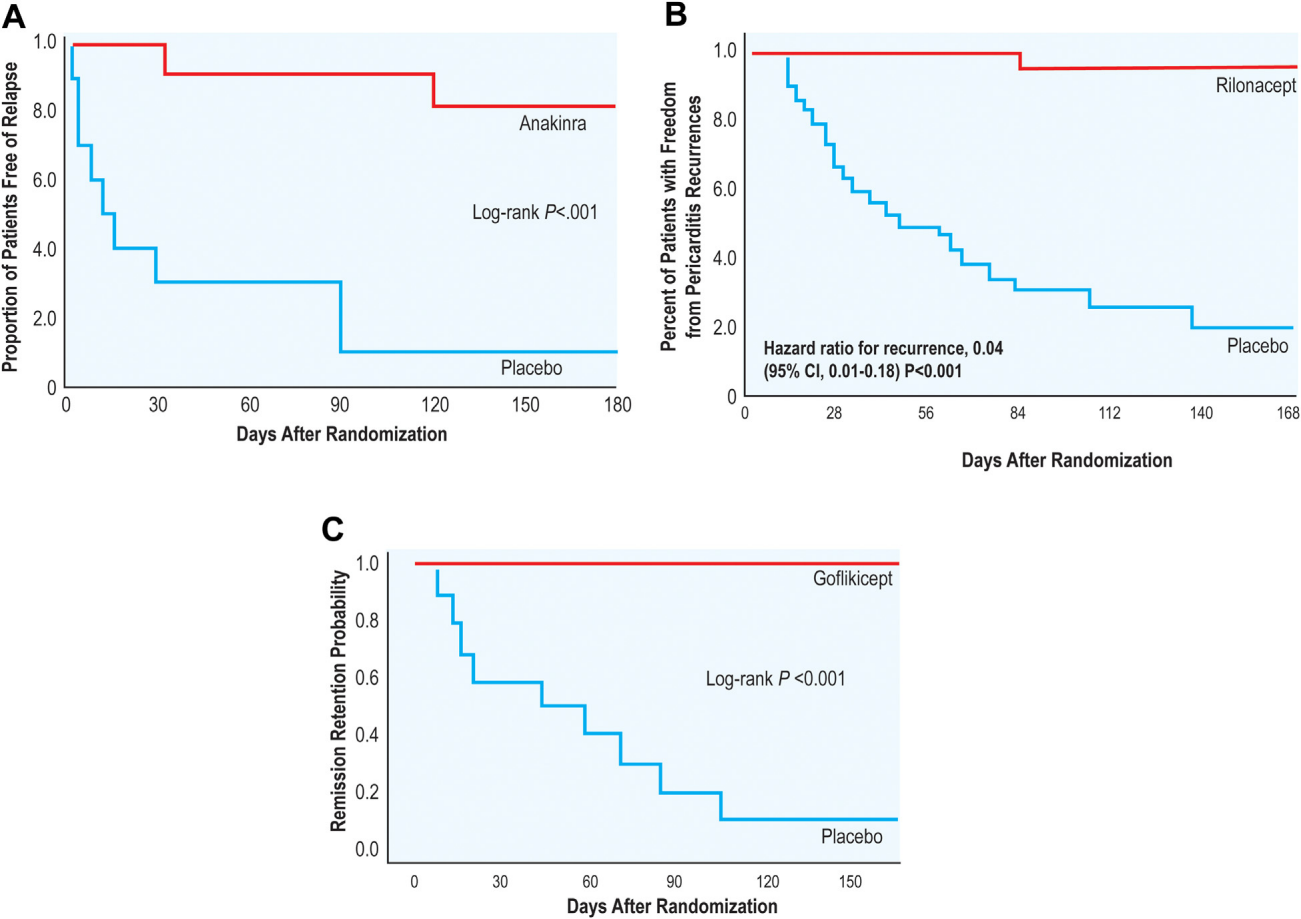
**Time to Pericarditis Recurrence in IL-1 Blocker Trials** AIRTRIP (AnakinraTreatment of Recurrent Idiopathic Pericarditis) (A), RHAPSODY (Rilonacept Inhibition of Interleukin-1 Alpha and Beta for Recurrent Pericarditis: A Pivotal Symptomatology and Outcomes Study) (B), and Goflikicept (C) clinical trials demonstrated a substantial reduction in pericarditis recurrence compared to placebo following randomized withdrawal (Klein et al^59^). IL = interlukin.

Recurrent pericarditis may be driven by an autoinflammatory phenotype. In patients who are infection-negative, steroid-dependent and nonresponsive to colchicine, as in the case presented, typically demonstrate this phenotype. IL-1 blockers have revolutionized the treatment of recurrent pericarditis and provide a paradigm shift in management. Figure 3 shows the 3 agents with most promise: rilonacept, anakinra, and goflikicept.^60^ Of these, rilonacept is currently approved by the Food and Drug Administration for treatment of recurrent pericarditis.^59^ Rilonacept is a chimeric fusion protein that functions as a decoy receptor for IL-1α and IL-1β, thereby effectively removing them from the circulation in their active form.^61^ Inhibition of these key effector cytokines blunts the autoinflammatory cascade that drives recurrent pericarditis. A pilot study of 25 patients with recurrent pericarditis first showed its safety and effectiveness in providing resolution of pain and inflammation, reduction in corticosteroid dependence, and improvement in quality of life.^62^ The drug was subsequently studied in the RHAPSODY (Rilonacept Inhibition of Interlukin-1 Alpha and Beta for Recurrent Pericarditis: A Pivotal Symptomatology and Outcomes Study).^59^ In this multicenter randomized withdrawal trial, 86 patients with at least 2 prior recurrences of pericarditis, elevated CRP and active chest pain requiring NSAIDs, colchicine or glucocorticoid therapy were enrolled in a 12-week run-in period where rilonacept was initiated and other medications were discontinued. Of these, 61 patients met a prespecified response criteria (CRP <0.5 mg/dL and pain score <2) and were randomized 1:1 to continue rilonacept or placebo. Recurrence only occurred in 2 patients (7%) in the rilonacept group compared to 23 patients (74%) in the placebo group, who had a median time of 8.6 weeks to recurrence. Furthermore, rilonacept provided rapid relief of pain and improvement of CRP within 7 days. The loading dose of the medication was 320 mg followed by weekly injection of 160 mg with a median duration of therapy of 9 months. Common adverse events were injection site reactions and upper respiratory tract infections.^59^ While the medication has a long half-life, stopping remains a challenge. A long-term extension study of RHAPSODY showed that continuation of rilonacept beyond 18 months resulted in continued treatment response while suspending medication use at 18 months was associated with risk for pericarditis recurrence, though the sample size was small in this study.^63^ Currently, rilonacept therapy is recommended for at least 2 years. At that time, clinicians should engage in shared decision-making with patients on whether to taper the medication gradually or to stop at once. Usually a background of colchicine therapy may be added when rilonacept is being tapered or stopped due to the high recurrence rate. Overall, rilonacept is a paradigm shifting medication in the treatment of recurrent pericarditis as it not only offers a rapid resolution of symptoms but also significantly lowers risk of recurrence. Anakinra is an IL-1α and IL-1β receptor antagonist and diminishes the autoinflammatory cascade. AIRTRIP (Anakinra-Treatment of Recurrent Idiopathic Pericarditis) was a randomized withdrawal trial of 21 patients who had at least 3 prior recurrences of pericarditis, CRP elevation and colchicine resistance and corticosteroid dependence.^64^ Patients received anakinra at a 2 mg/kg daily injection (maximum 100 mg/d) for 2 months. During this period, NSAIDs and corticosteroids were tapered though 57% of patients still continued colchicine. Patients who demonstrated resolution of pericarditis were randomized 1:1 to anakinra or placebo. Recurrence of pericarditis only occurred in 2 patients on anakinra and 9 patients on placebo over a median duration of 14 months.^64^ Common adverse reactions were transient injection site reactions, and asymptomatic transaminase elevation. A subsequent analysis of 224 patients with steroid dependence and colchicine resistance (IRAP registry) showed a 6-fold reduction in pericarditis recurrence and reduction of corticosteroid use from 80% to 27% following a median treatment duration of 6 months.^65^ While limited data are available regarding optimal tapering regimen of anakinra, general recommendations are to taper slowly. One possible approach is to begin tapering following 6 to 12 months of successful therapy with resolution of clinical symptoms and inflammatory markers. Anakinra can be tapered by 100 mg/wk every month until a 300 mg/cumulative weekly dose is achieved and then further reduction by 100 mg/wk every 2 to 3 months.^66^ Goflikicept is a new agent that functions as a heterodimer fusion protein trapping IL-1α and IL-1β to mitigate effector cytokines.^67^ In a study design similar to RHAPSODY and AIRTRIP, 22 patients were enrolled and 20 were randomized with idiopathic recurrent pericarditis, at least 1 prior recurrence and an active disease state whom were treated with goflikicept for a run-in period followed by randomization and withdrawal. A positive response was measured as improvement of chest pain, CRP level and pericardial effusion compared to initiation. Not a single patient in the goflikicept group experienced a recurrence compared to 90% of patients in the placebo group.^67^ Loading dose of the medication was 160 mg followed by 70 mg 1 week later, 80 mg 1 week after, and for every 2 weeks thereafter as a maintenance dose. Duration of goflikicept therapy and details about proper tapering regimen need further investigation, but golflikicept shows promise as a novel therapeutic agent.

## Proposed diagnostic and treatment approach

Recurrent pericarditis can manifest as either a noninflammatory or autoinflammatory phenotype. Central Illustration outlines our proposed imaging-guided approach for treatment of recurrent pericarditis. Following first recurrence, patients should be treated with a prolonged duration of high-dose NSAIDs (weeks to months) and colchicine (>6 months). If a patient experiences a second recurrence while on high-dose anti-inflammatory medications or while on a taper of these medications, then CMR and serum inflammatory markers should be obtained. A combination of elevated serum inflammatory markers (CRP >1 mg/dL) and presence of pericardial LGE on CMR indicates an autoinflammatory phenotype. Prompt utilization of CMR aids clinicians to identify the appropriate phenotype early, risk-stratify patients based on their likelihood of recurrence, and select appropriate treatment. Based on our practice, patients with an autoinflammatory phenotype may benefit from earlier initiation of IL-1 inhibitors (ie after second recurrence) as opposed to corticosteroids given the high efficacy of IL-1 inhibitors in corticosteroid-dependent patients. Follow-up CMR imaging may be obtained at least 6 to 12 months and if LGE is improved, then this may indicate less inflammation and neovascularization. Tapering or discontinuing treatment should be based on shared decision-making with patients, based on clinical symptoms, CMR findings, and inflammatory markers.

**Central Illustration:**
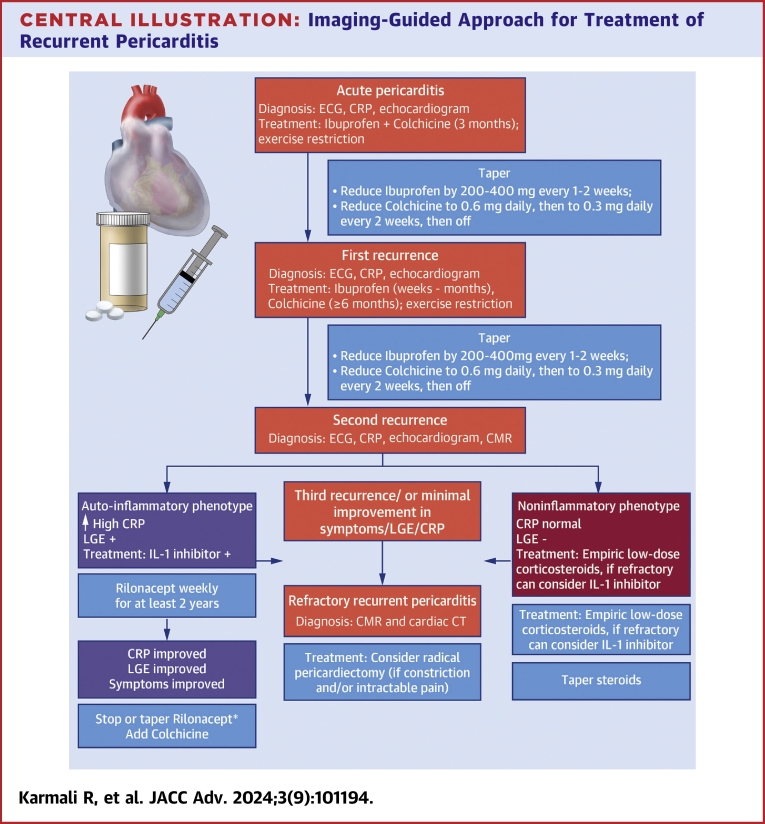
**Imaging-Guided Approach for Treatment of Recurrent Pericarditis** Following identification of phenotype, a step-wise escalation in approach of therapies is ideal. In the auto-inflammatory phenotype, IL-1 blockers may be considered at an earlier juncture. At our center, ibuprofen is the preferred NSAID and Rilonacept is the preferred IL-1 blocker. ∗Limited data are available regarding stopping or tapering strategy for Rilonacept. Clinicians should engage in shared-decision making with patients on whether to taper the medication gradually or to stop at once following completion of 2 years of treatment. CMR = cardiac magnetic resonance; CRP = C-reactive protein; CT = computed tomography; LGE = late gadolinium enhancement; other abbreviations as in Figures 1 and 3.

On the other hand, if a patient exhibits a noninflammatory phenotype (low to normal inflammatory markers and absence of significant LGE on initial CMR) in the presence of definite pericarditis (seen, for example, via pericardiocentesis), then they should be treated empirically with corticosteroids (>0.25-0.5 mg/kg/d) in a background of colchicine. Corticosteroids should be tapered slowly (5-10 mg every 1-2 weeks) while colchicine is continued. An echocardiogram should be obtained each time a recurrence is suspected to evaluate for new or worsening pericardial effusion or development of constrictive physiology. If a patient develops signs of constriction and heart failure, then a cardiac CT should be obtained to evaluate for pericardial calcification burden. Calcified constrictive pericarditis represents an advanced stage of the disease process that is unlikely to respond to medical treatment and at this stage, a radical pericardiectomy should be considered.

## Conclusions and future investigations

IL-1 blockers have paved the way for a promising future in the treatment of recurrent pericarditis. A recently published international position statement emphasizes the importance of mutimodality cardiac imaging and novel therapeutics in pericardial disease treatment.^68^ Key differences from the RHAPSODY, AIRTRIP, and Gofikicept trials raise important questions for future investigations. First, given number of recurrences at time of enrollment in each trial (ie after first vs second vs third recurrence), at which stage can IL-1 blockers be initiated in treatment? Second, since serum inflammatory markers can be suppressed on treatment, can imaging-guided therapy inform decisions about IL-1 duration? A major concern for patients is inability to exercise with recurrent pericarditis. Recent observations suggest that patients can increase their activity while on IL-1 blockers but further clinical trial data are necessary. Answering these questions will help further our understanding and management of the clinical challenge that is recurrent pericarditis.

## Funding support and author disclosures

This work was supported by 10.13039/100007312Cleveland Clinic Foundation. Dr Klein has received research funding from 10.13039/100016492Kiniksa Pharmaceuticals, Ltd and Cardiol Therapeutics; and has served on scientific advisory boards for 10.13039/100016492Kiniksa Pharmaceuticals, Ltd, Cardiol Therapeutics, and 10.13039/100004319Pfizer, Inc. Dr Cremer has received research grants from Kiniksa Pharmaceutics and Novartis, and is on advisory boards for Kiniksa Pharmaceutics and Swedish Orphan Biovitrum. All other authors have reported that they have no relationships relevant to the contents of this paper to disclose.
